# How to manage delayed high-grade kidney trauma on pediatric and its complications: A case report

**DOI:** 10.1016/j.ijscr.2025.111067

**Published:** 2025-02-17

**Authors:** Nadya Rahmatika, Soetojo Wirjopranoto, Bagus Wibowo Soetojo, Yufi Aulia Azmi, Antonius Galih Pranesdha Putra, Kevin Muliawan Soetanto

**Affiliations:** a Faculty of Medicine, Wijaya Kusuma University, Surabaya, Indonesia; b Department of Urology, Faculty of Medicine Universitas Airlangga – Dr. Soetomo General Academic Hospital, Surabaya, Indonesia; c Departement of Orthopaedic and Traumatology, Faculty of Medicine, Universitas Airlangga – Dr. Soetomo General Academic Hospital, Indonesia; d Department of Health Sciences, University of Groningen, University Medical Center Groningen, Groningen, the Netherlands; e Department of Immunology, Faculty of Medicine Siriraj Hospital, Mahidol University, Bangkok, Thailand

**Keywords:** High-grade renal trauma, Urinoma, Pediatric, Case report, Infection

## Abstract

**Introduction and importance:**

Trauma results in more deaths in childhood than all other causes combined, one of which is high kidney trauma. This case report presents minimally invasive management of high-grade kidney trauma in pediatrics.

**Case presentation:**

A 5-year-old boy was referred on day 10 for blunt abdominal trauma. The complaint was intermittent high fever and right back pain after falling from the bike. There are no open wounds. A 9 × 7 cm cystic mass is palpable in the upper right abdominal quadrant. The results of the Abdominal Computed Tomography (CT) scan revealed AAST Grade V kidney trauma with a gap of 3.9 cm, free perirenal fluid on the right side inside the *Gerota fascia*, and a fluid size of 9.7 × 6.7 × 7.4 cm, with a volume of 256 ccs (HU 8 to 12). Retrograde pyelography (RPG) was performed on the right kidney, contrast extravasation was found, a Double J (DJ) stent was inserted, and percutaneous urinal drainage was performed under ultrasound guidance.

**Clinical discussion:**

A CT scan can be used as a detection tool for cases of neglected high-grade kidney trauma. Minimal invasive management can be performed when the patient is in stable condition. Haematuria, fever, and urinoma can be found as a complication.

**Conclusion:**

In cases of blunt abdominal trauma in children, there should be suspicion of kidney trauma until the diagnosis is established. If there is a urinoma, installing a DJ Stent and percutaneous drainage is an option.

## Highlights

• Minimal Invasive Management due to neglected high-grade renal trauma complication on pediatric.
• The importance of complete examination for suspect renal trauma.
• Pediatric high-grade renal trauma needs comprehensive examination and management.

## 1 Introduction

Trauma is believed to be the leading cause of death worldwide. The kidneys are the most common place, representing over half of all urinary tract injuries. Kidney trauma is divided into blunt and translucent, with blunt trauma accounting for 90 % of kidney injuries. Kidney injury occurs in up to 5 % of trauma patients and accounts for 24 % of traumatic abdominal dense organ injuries. [1]. Today, trauma results in more deaths in childhood than all other causes combined [2]. Children are at high risk of kidney injury due to blunt trauma due to their anatomy [3]. Trauma is the leading cause of morbidity and mortality in children, with about 3 % of children assessed in children's hospital trauma departments to have trauma [4].

Most children with blunt kidney trauma have multi-organ injuries to the spleen, liver, head, and bones [5]. Early complications include urinoma, delayed bleeding, urinary fistulas, abscesses, and hypertension. Advanced complications after kidney trauma include hydronephrosis, arteriovenous fistula, pyelonephritis, calculus formation, and delayed hypertension. Urinoma is the most common complication of kidney trauma, and delayed bleeding usually occurs within 1–2 weeks of injury [6]. Urinomas are a collection of chronic urine extravasation that is encased in a capsule [7]. Most complications can be treated non-operatively, percutaneously, and endourology [8].

Treatment varies according to etiology and can range from vigilant waiting to percutaneous drainage to, in rare cases, a nephrectomy [9]. Non-operative management has been reported to be safe and effective in these cases, even in patients with hemodynamically unstable high-grade kidney injury in some literature. However, there is still debate about the treatment of patients with high-grade kidney injury [1]. The optimal management of kidney injury has been debated for a long time. Operative management for high-grade kidney injury has not been shown to improve recovery [10]. The scarcity of kidney trauma limits the research and the strength of evidence-based guidelines. Although kidney injury management has shifted to a non-operative approach, nephrectomy remains the most common intervention for high-grade renal trauma (HGRT) [11]. According to the guidelines, death can be avoided with proper treatment, and hemodynamic stability is a decisive factor [4]. Based on this background, this case report reports a minimally invasive management for neglected high-grade kidney trauma in pediatrics. Reports of this case have been reported in line with the SCARE Guidelines [12].

## 2 Case presentation

A 5-year-old boy was referred to Our emergency room (ER) and diagnosed with neglected blunt abdominal trauma. The patient's main complaint was intermittent high fever accompanied by intermittent right back pain. The fever and back pain with visual analog score (VAS 6) had been felt for one week and worsened 2 days before hospital admission. The pain has been felt intermittently since falling from the bike 10 days ago and was also followed by intermittent haematuria. The patient's right waist hit a brick when falling off the bike. The patient performed normal activities since there were no hematomas or open wounds. The parents with a low education level only give antipyretics if the patient has had a fever and pain and routinely massage the waist at night when it has been painful for the past 10 days.

**Fig. 1:**
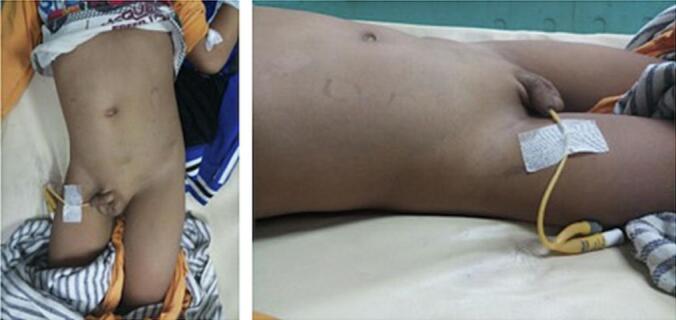
Clinical picture of the patient.

**Fig. 2:**
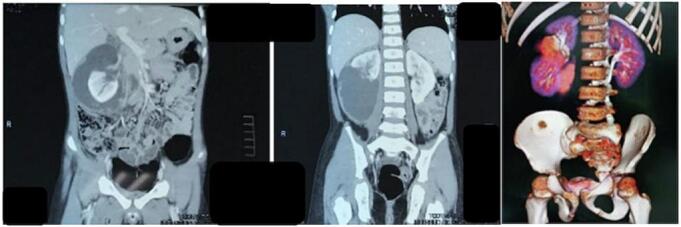
Abdominal CT scan of the patient.

From the patient's physical examination in the ER, the body temperature was 41C, and a cystic mass measuring 9 × 7 cm was palpable at the upper right abdominal quadrant (Fig. 1). The patient's laboratory results showed hemoglobin 8.9 g/dL, leukocytes 46.58 uL, increasing Serum Creatinine (SC) 2.04 mg/dL and CRP increased to 4.34. An Abdominal Computed Tomography (CT) scan results revealed AAST Grade V renal trauma with a gap of 3.9 cm, free perirenal fluid on the right side inside the *Gerota Fascia*, and a urine size of 9.7 × 6.7 × 7.4 cm, with a volume of 256 ccs (HU 8 to 12) (Fig. 2).

**Fig. 3:**
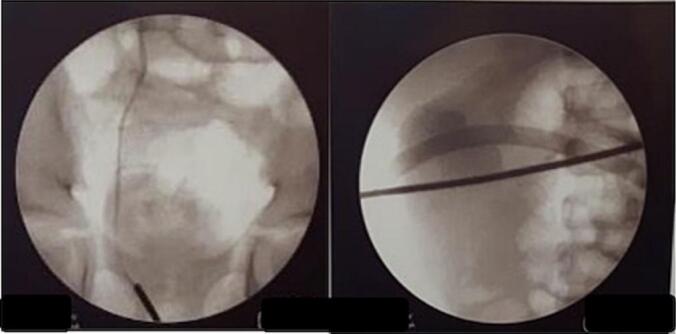
Retrograde pyelography (RPG) of the patient.

An empirical antibiotic, ceftriaxone 1350 mg/24 h intravenous (iv), was given. After the patient was in good condition, we performed a cystoscopy. There were intra bladder blood clots during cystoscopy; it was evacuated with a total volume of 20 cc blood clots; both ureteral openings were normal. Retrograde pyelography (RPG) was performed on the right kidney, and contrast extravasation was found (Fig. 3). A 4.6 fr Double J (DJ) stent was inserted, and percutaneous drainage of the urinoma was performed. Drainage of the urinoma was performed under ultrasound guidance, with the insertion of pigtails, resulting in an initial drainage of 150 mL of urinoma. The pus culture results showed *Escherichia Coli* was sensitive to ceftriaxone.

The following day after surgery, the patient was in good general condition, with no complaints, good vital signs, 700 cc/24-h urine production, and 300 cc/24-h percutaneous drainage production. On Day 2 post-operative, the total percutaneous drainage volume was 250 cc/24 h. On Day 3 post-operative, the total percutaneous drainage volume was 200 cc/24 h, the leucocyte decreased to 10 uL, and CRP was normal. He was discharged from the hospital on Day 4 post-operative. The pigtails were removed 1 week after minimal production on a polyclinic visit. The DJ Stent was removed 4 weeks after the insertion, with no clinical sign of hematuria, and the ultrasonography (USG) evaluation showed no urinoma. An abdominal CT Scan with contrast and a renal scintigraphy evaluation was offered to the parents, but they refused because of the cost. Monthly routine urology polyclinic visits 6 months after hospital discharge showed normal SC, 1.0 mg/dL, and no hypertension on vital signs.

## 3 Discussion

This case report highlights the importance of vigilance and suspicion of abdominal blunt trauma in children. Abdominal blunt trauma in children is pressure, pressure, or slowing down in the abdomen. It is the third most common cause of death from trauma in children, but it often goes unrecognized [13]. The abdomen is the third anatomical region affected in children, after the head and limbs, and accounts for about 25 % of major trauma [14]. Abdominal blunt trauma in childhood presents a diagnostic and therapeutic challenge that has tested clinical and radiological skills [15].

The administration of antibiotics is necessary in cases of neglected high-grade kidney trauma, especially if there is indeed leukocytosis or sepsis. IV broad-spectrum antibiotics should be used if there is a suspicion of damage to the collection system and urine leakage [16]. Antibiotics are essential for treating sepsis, but indiscriminate use can increase resistance worldwide. The selection of appropriate empirical therapies considers the location of infection, local epidemiology, host comorbidities, and recent antibiotic exposure. Culture results and clinical improvement will guide de-escalation and duration of treatment [17]. Surgical site infections are a common complication after abdominal trauma and are associated with increased morbidity, mortality, and length of hospital stay [18].

Minimally invasive management in pediatrics can be used as an option, namely by percutaneous drainage and the installation of DJ Stents. Safety percutaneous drainage can be done on children and the outcome shows good results. Percutaneous drainage management for urinoma diversion, avoiding sepsis complications. A catheter or needle is inserted under the skin to drain a fluid collection using percutaneous drainage, a minimally invasive technique guided by imaging. Percutaneous drainage may promote healing and prevent or treat abscesses if perinephric fluid collection continues after ureteral stenting or percutaneous nephrostomy drainage. [19]. Percutaneous drainage, nephrostomy tubes, or open surgery may be necessary for larger urinomas in addition to treating the underlying cause. [20]. For patients undergoing radical cystectomy and urinary diversion, percutaneous drainage is a safe and efficient method of collecting fluids after surgery. [21]. Other studies found minimally invasive treatments such as angioembolization or ureter stents were seen in about 5 % of all cases, with 10 % to 15 % for 4 to 5-degree injuries. Nephrectomy for pediatric kidney trauma is rare and likely occurs in about 5 % of patients treated [4].

In children with blunt abdominal trauma, with a history of falling/being hit with a sharp object or blunt object in the lumbar area, the suspicion of kidney trauma must remain and the patient should be taken to the hospital for a radiological examination. The primary diagnosis of renal trauma is based on computed tomography (CT) with contrast, which is indicated in all stable patients with severe hematuria and patients presenting with microscopic hematuria and hypotension. In addition, CT should be performed when the mechanism of injury or physical examination findings indicate the presence of renal injury (e.g. rapid deceleration, rib fractures, pelvic, and any penetrating injury to the abdomen, pelvis, or lower chest) [22]. Computerized tomography can help assess kidney injury and additional organ injury quickly [23]. Moreover, CT with contrast (CECT) of the abdomen and pelvis can provide a detailed assessment of kidney injury, including kidney bruises, lacerations, hematomas, and active bleeding [24].

Postoperative management is carried out by checking vital signs, especially blood pressure, which needs to be considered when in the room while the patient is being treated in the hospital, as well as the total production of percutaneous drainage. Hypertension is a rare complication of blunt kidney trauma, with a higher risk in cases of major kidney trauma. The progression of hypertension after blunt kidney trauma can be heterogeneous, with a time of manifestation that spans between a few days after the accident and several years after [25]. Standard approaches used to treat blunt kidney damage include monitoring vital signs and hemoglobin/hematocrit levels [4].

In these cases, the patient can be treated minimally invasively. Pediatric kidney trauma can be successfully managed non-surgically in more than two-thirds of cases in middle-income countries. High-grade kidney injury does not predict the need for surgery or nephrectomy and can be managed non-surgically [26]. Conservative management is recommended for high-grade renal trauma in hemodynamically stable patients. High-grade vascular injury is more severe than parenchymal injury and correlates with poorer renal functional outcomes. [27]. After the exclusion of hemodynamic instability and continuous bleeding, conservative treatment is successful in 80 % of patients. Internal stenting with or without percutaneous drainage is indicated if there is a progressive urinoma. Angioembolization is successful in certain cases. [28].

Post-opeative evaluation can be done with a CT scan, MRI, or renal scintigraphy. Another alternative can be an ultrasound examination, blood pressure monitoring and serum creatinine for 6 months post-incident. Ultrasound can be used to determine post-injury anatomy without additional ionizing radiation. Kidney scintigraphy can show how well the kidneys are functioning and how they look. This test can be used to document functional recovery after kidney injury. CT is very useful in evaluating traumatic injuries to the kidneys with pre-existing abnormalities. Blood pressure monitoring is recommended annually to rule out reconstructive hypertension. All cases of kidney trauma should be monitored with regular blood pressure recording for at least the first year after injury. Arterial hypertension can occur as a complication of kidney trauma with an incidence of up to 40 % [29].

## 4 Conclusion

In cases of blunt abdominal trauma in children, there should be suspicion of kidney trauma until the diagnosis is established. If there is a urinoma, the installation of a DJ Stent and percutaneous drainage can be done.

## Ethical approval

Ethical approval for this study was provided by Health Research Ethics Committee of the hospital.

## Author contributions

Nadya Rahmatika: Conceptualization, Data Curation, Writing-Original draft preparation

Bagus Wibowo Soetojo: Conceptualization, Data Curation, Writing-Original draft preparation

Soetojo Wirjopranoto: Conceptualization, Methodology, Data Curation, Investigation, Writing-Original draft preparation, Supervision, Validation

Yufi Aulia Azmi: Conceptualization, Methodology, Data Curation, Investigation, Writing-Original draft preparation, Supervision, Validation

Antonius Galih Pranesdha Putra: Writing-Original draft preparation, Writing-Reviewing, and Editing

Kevin Muliawan Soetanto: Writing-Original draft preparation, Writing-Reviewing, and Editing

## Funding report

No financial contributions were made by the authors to this study.

## Declaration of competing interest

The author stated that there was no conflict of interest.

## Data Availability

The research outlined in the article does not make use of any data.
